# Impact of Duodenal Papilla Morphology on the Success of Transpancreatic Precut Sphincterotomy

**DOI:** 10.3390/jcm13226940

**Published:** 2024-11-18

**Authors:** Yi-Peng Chen, Yi-Jun Liao, Yen-Chun Peng, Chun-Fang Tung, Hsin-Ju Tsai, Sheng-Shun Yang, Chia-Chang Chen

**Affiliations:** 1 Division of Gastroenterology and Hepatology, Department of Internal Medicine, Taichung Veterans General Hospital, Taichung 407219, Taiwan; great800700@vghtc.gov.tw (Y.-P.C.); s19001029@gmail.com (Y.-J.L.); pychunppp@gmail.com (Y.-C.P.); et@vghtc.gov.tw (C.-F.T.); a9194024@hotmail.com (H.-J.T.); yansh@vghtc.gov.tw (S.-S.Y.); 2 Department of Post-Baccalaureate Medicine, College of Medicine, National Chung Hsing University, Taichung 402202, Taiwan; 3 School of Medicine, National Yang Ming Chiao Tung University, Taipei 112304, Taiwan; 4 Ph.D. Program in Translational Medicine, National Chung Hsing University, Taichung 402202, Taiwan; 5 Institute of Biomedical Sciences, National Chung Hsing University, Taichung 402202, Taiwan

**Keywords:** transpancreatic precut sphincterotomy, endoscopic retrograde cholangiopancreatography, papillary cannulation, duodenal papillary morphology

## Abstract

**Background**: This study aimed to evaluate whether the morphology of the duodenal major papilla is linked to transpancreatic precut sphincterotomy (TPS) failure. **Methods**: We conducted a retrospective review of patients who underwent endoscopic retrograde cholangiopancreatography (ERCP) at our institution. The inclusion criteria involved patients with a naïve major duodenal papilla who required TPS due to difficult biliary cannulation. Papilla morphology was classified using Haraldsson’s system, as follows: regular (Type 1), small (Type 2), protruding or pendulous (Type 3), and creased or ridged (Type 4). The analysis focused on identifying risk factors for TPS failure and related complications. **Results**: A total of 103 cases were analyzed, with an overall TPS success rate of 85.44%. There were no significant differences in age, gender, ERCP indications, or the prevalence of juxtapupillary diverticula across the four papilla types. The TPS failure rates by papilla type were Type 1 (10.53%), Type 2 (0%), Type 3 (16.67%), and Type 4 (28%). Type 4 papilla had a significantly higher failure rate compared to Type 1 and Type 2 in the univariate analysis (*p* = 0.028), but this was not statistically significant in the multivariate analysis (*p* = 0.052). Age emerged as an independent risk factor for TPS failure. **Conclusions**: Duodenal papilla morphology may influence the success rate of TPS, with advanced age being a key risk factor for failure. Identifying high-risk factors such as Type 4 papilla and older age can help endoscopists adjust their techniques early, potentially improving outcomes and minimizing complications.

## 1. Introduction

Endoscopic retrograde cholangiopancreatography (ERCP) is essential for the diagnosis and management of biliary and pancreatic disease. Successful cannulation of the biliary duct is a critical component of ERCP, with reported failure rates for biliary cannulation ranging between 5% and 15% [1,2,3]. The definition of difficult biliary cannulation has varied widely across studies, reflecting differences in the criteria such as the number of attempts, duration of cannulation, and reliance on alternative techniques. This variability has led to inconsistencies in identifying cases that may require advanced cannulation methods or alternative approaches [4,5,6]. According to international consensus recommendations, difficult biliary cannulation is defined by the inability to achieve selective biliary access using conventional ERCP techniques within 10 min or after up to five cannulation attempts, or by the inability to access the major papilla [6].

Numerous methods have been developed to overcome difficult biliary cannulation, including approaches such as the double-guidewire technique, transpancreatic precut sphincterotomy (TPS), needle-knife papillotomy, fistulotomy, and the rendezvous technique. These techniques aim to enhance access and increase the success rate of selective biliary cannulation in challenging cases [7,8,9]. TPS, initially introduced by Goff in 1995 [10], entails a careful incision of the septum located between the common bile duct (CBD) and the pancreatic duct (PD) when the guidewire has passed into the PD. Using a standard sphincterotome over the guidewire, the instrument is directed through the septum and angled towards the bile duct, thus facilitating access and cannulation of the CBD [10,11,12,13]. The success rate of CBD cannulation following TPS ranges from 85% to 100% [12]. Previous studies have reported TPS as a safe and effective method for achieving biliary access in cases of difficult cannulation, especially when performed by skilled and experienced advanced endoscopists [14].

Haraldsson’s classification system, which divides papilla into four types (regular, small, protruding, and creased), has been valuable in evaluating papillary characteristics. In our previous research, we found that duodenal major papilla morphology, as classified by Haraldsson, can impact biliary cannulation and related complications during ERCP [15]. However, its application specifically in TPS has not been adequately studied. Understanding how papilla morphology influences TPS success could help endoscopists tailor their approach during difficult cannulation cases, potentially improving outcomes and minimizing complications.

This study aims to examine if the morphology of the duodenal major papilla contributes as a risk factor to the failure of TPS and the incidence of related complications, addressing an important gap in the literature. By doing so, we seek to provide a foundation for better decision making in clinical practice, particularly in the management of patients with challenging papillary anatomy.

## 2. Materials and Methods

### 2.1. Data Source

This study was conducted at Taichung Veterans General Hospital, a tertiary care center with an annual ERCP volume ranging from 750 to 1000 cases. We performed a retrospective analysis of medical records for patients who underwent ERCP at our institution. The inclusion criteria focused on patients for whom TPS was the first advanced cannulation technique employed for challenging bile duct access during therapeutic ERCP, specifically with a naïve major duodenal papilla.

The exclusion criteria encompassed diagnostic ERCP procedures (e.g., involving only cholangiogram procedure without desired duct cannulation, which were infrequent in our hospital and were excluded from this investigation); individuals below 18 years of age; cases involving pancreatic management (e.g., primary interest in the pancreatic duct); periampullary diverticulum (PAD) Type 1; a known factor complicating papilla classification and cannulation; cases with involvement of the papilla by a tumor; instances where papilla classification was hindered due to factors such as swelling of the duodenal mucosa, deformities, or ulceration on the surface of the papilla; and a pre-cut procedure using a needle knife was employed as the initial advanced cannulation technique.

### 2.2. Cannulation Process

At our institution, ERCP procedures were carried out by skilled endoscopists, each with experience performing more than 1000 ERCPs. Trainees, including fellows and junior practitioners with experience in under 1000 ERCPs, were actively involved in these procedures. Trainees need to have at least two years of endoscopy experience and observe in the ERCP room for 3 to 6 months. After obtaining approval from the senior endoscopists, trainees were allowed to start the bile duct cannulation. If, at any stage of the procedure, the supervising endoscopist determined that the trainee could not successfully achieve cannulation, they immediately took control of the cannulation process. Trainees in our study had no specific time constraints for their cannulation efforts, with neither minimum or maximum time limits imposed. This approach applied to both bile duct and pancreatic duct cannulation attempts, allowing trainees the flexibility to focus on technique rather than adhering to strict time guidelines.

For biliary cannulation, a standard sphincterotome was used with guidewire assistance (e.g., TRUEtome, Boston Scientific Corporation, Marlborough, USA; RotaCut Medi-Globe GmbH, Bavaria, Germany), alongside guidewires such as the Jagwire, Boston Scientific Corporation, Marlborough, USA and VisiGlide 2, Olympus Medical Systems Corp, Tokyo, Japan. When the guidewire did not advance into the bile duct, a minimal contrast injection was carefully applied to enhance visualization of the duct endpoint, facilitating successful cannulation. If difficult cannulation occurred, we tried advanced techniques like TPS, a needle-knife or double-guidewire technique. In this study, we included patients who underwent TPS as the initial advanced cannulation technique to overcome difficult biliary cannulation. If the TPS didn’t succeed, we might try an alternative pre-cut procedure involving a needle-knife or a double-guidewire technique. Patients who underwent the needle-knife procedure as an initial approach were excluded from the analysis.

The level of sedation for ERCP varies based on the patient condition and institutional guidelines. Moderate sedation is generally appropriate for stable patients who are able to manage mild discomfort, allowing them to respond to verbal prompts while maintaining cardiovascular stability. For more challenging or extended ERCP procedures, particularly those involving difficult cannulation, deep sedation is preferred. This approach minimizes patient movement and enhances comfort, though it requires close monitoring, as patients are less responsive to stimuli yet continue to breathe autonomously.

### 2.3. Data Collection

We performed a retrospective review of medical records for patients who underwent ERCP. In our institution, we carefully recorded information about the patients who had undergone ERCP procedures, noting down different details about the process. At least three photos were taken during the procedure, as follows: one at the start, the second one when the device makes contact with the duodenal major papilla, and the final one upon successful guidewire placement in the CBD. Data collected included the cannulation time, defined as the duration from the initial attempt to the successful guidewire placement in the CBD. Other recorded factors included the number of guidewire entries into the main pancreatic duct, whether pancreatic duct injection was performed, and the outcome of cannulation (success or failure). Additionally, we documented the use of various procedures, such as needle-knife sphincterotomy, standard sphincterotomy, endoscopic papillary balloon dilation (EPBD), stone extraction, lithotripsy, endoscopic retrograde biliary drainage (ERBD), and endoscopic retrograde pancreatic duct drainage (ERPD).

**Figure 1:**
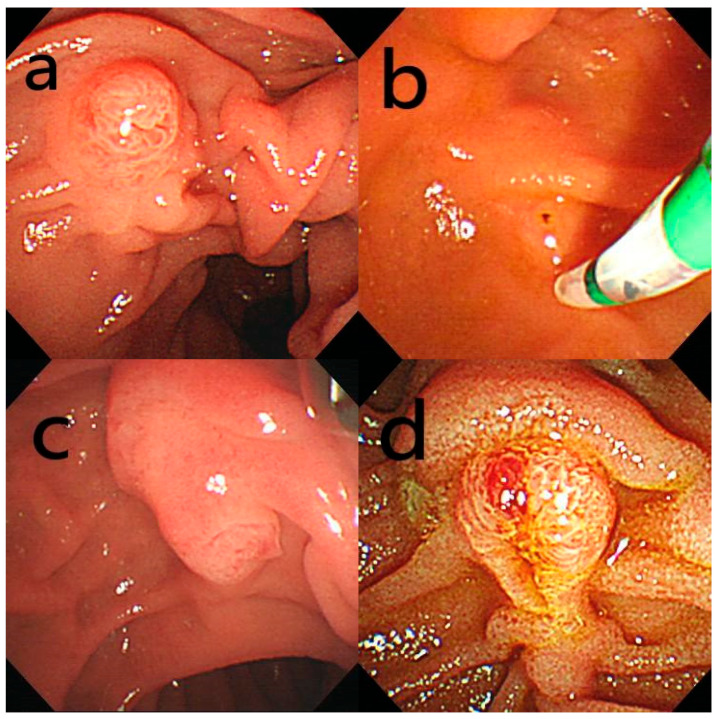
Endoscopic classification of papillae: (**a**) Type 1—regular papilla, displaying a “classic appearance” without distinctive features; (**b**) Type 2—small papilla, often appearing flat, with a diameter no larger than 3 mm (approximately 9 Fr); (**c**) Type 3—protruding or pendulous papilla, which noticeably extends or bulges into the duodenal lumen, or may hang down with a downward-oriented orifice; (**d**) Type 4—creased or ridged papilla, where the ductal mucosa appears to extend distally from the papillary orifice, forming either a ridge or a crease.

In this study, we employed Haraldsson’s classification to classify the major papillae into four categories: regular (Type 1), small (Type 2), protruding or pendulous (Type 3), and creased or ridged (Type 4). Type 1, or a regular papilla, has a classic appearance without distinct characteristics. Type 2, termed a small papilla, is generally flat with a diameter no larger than 3 mm (approximately 9 Fr). Type 3, known as a protruding or pendulous papilla, visibly extends or bulges into the duodenal lumen and may hang downward, with the orifice positioned in a downward-facing direction. Finally, Type 4, called a creased or ridged papilla, has ductal mucosa that appears to extend outward from the papillary orifice, forming either a pronounced ridge or a crease (Figure 1). Endoscopic images from all ERCP procedures were collected, grouped by patient, and reviewed by two endoscopists involved in the study. Each endoscopist independently categorized the papilla type for each set of images without knowledge of the patient’s other clinical information. In cases where classifications differed, a third endoscopist involved in the study was consulted to reach a final consensus.

**Figure 2:**
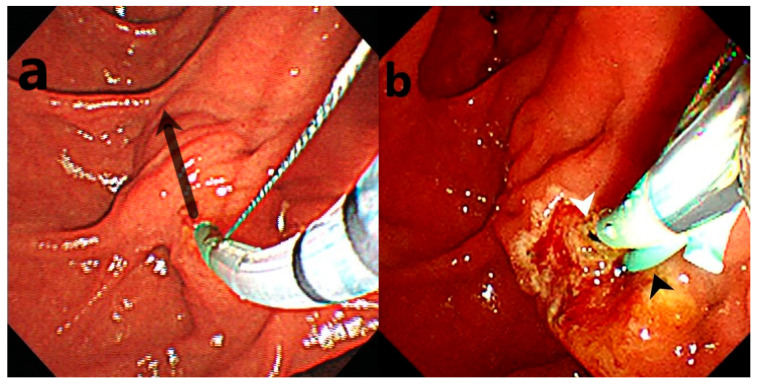
Illustration of the transpancreatic precut sphincterotomy (TPS) procedure: (**a**) a guidewire was positioned within the pancreatic duct, and then the septum was incised with a sphincterotome, extending the cut from the pancreatic duct toward the axis of the bile duct (arrow); (**b**) after placing a stent in the pancreatic duct (black arrow head), cannulation was directed toward the bile duct (white arrow head).

All ERCP procedures conducted within our hospital were undertaken on an inpatient basis. Subsequent to the ERCP, a comprehensive follow-up was conducted for all patients to evaluate both immediate and late adverse events until the point of discharge. Adverse events were systematically recorded in accordance with established guidelines [16,17]. According to ESGE guidelines, post-ERCP pancreatitis (PEP) is diagnosed when new or worsening abdominal pain occurs alongside amylase or lipase levels that are over three times the normal limit for more than 24 h post-ERCP, with either hospital admission or an extended stay required. Cholangitis is indicated by a fever exceeding 38 °C that lasts over 24 h, coupled with cholestasis. Bleeding is identified through symptoms like hematemesis or melena, or if hemoglobin levels drop by more than 2 g/dL. Perforation is confirmed by imaging that reveals the presence of gas or luminal contents outside the gastrointestinal tract [17]. Furthermore, demographic data, including age, gender, and indications for ERCP, of the patients were comprehensively documented. The TPS procedure is illustrated in Figure 2.

### 2.4. Statistical Methods

Statistical analyses were performed using SPSS (version 13.0; Chicago, IL, USA). A power analysis was conducted to ensure the adequacy of the sample size for detecting significant differences. This study builds upon our previous research [18] focusing on two distinct groups: group 1, characterized by a 46% failure rate of TPS for Type 4 papilla, and group 2, exhibiting a 13% failure rate in other papilla cases. By extending our prior research, Group 1 is anticipated to require approximately 17 cases to reach the desired 80% power, while group 2 is estimated to necessitate around 51 cases for a comparable statistical power. Categorical variables were analyzed using the Chi-squared test and Fisher’s exact test, while continuous variables were reported as median with interquartile range (IQR) and assessed through the Mann–Whitney U test and Kruskal–Wallis test. The interobserver agreement evaluation was assessed using kappa statistics (Table A1).

A linear regression analysis was employed to investigate factors associated with cannulation failure. Factors demonstrating significance (*p*  <  0.05) in the univariate analysis were further analyzed using multivariate analysis to identify independent predictive factors. Statistical significance was established with a *p*-value of less than 0.05. The study received approval from the Ethical Review Board of TCVGH (reference number: CE22198B; approval date: 25 May 2022).

## 3. Results

From September 2017 to June 2024, 107 patients underwent TPS as the initial advanced cannulation technique for difficult bile duct cannulation during therapeutic ERCP with a naïve major duodenal papilla. We excluded the following four cases: one with an unclassifiable papilla due to duodenal mucosa swelling, one with a papillary tumor, and two with stone impaction in the papilla. The final analysis included 103 ERCPs.

Demographic information for all patients is detailed in Table 1. The average age of the participants was 64 years, with 57% being male. The leading indication for performing ERCP was bile duct stones, accounting for 57 cases or 55% of the total, followed by malignancy-related bile duct obstruction, which occurred in 36 cases, representing 35%. Among the malignant cases, eight were diagnosed with bile duct cancer (22% of all 36 malignant obstructions), and 18 had pancreatic cancer (50%). The remaining 28% of cases involved non-biliary cancers, such as hepatocellular carcinoma and metastatic lymphadenopathy. The majority of papillae were identified as Type 1 (55.3%), while Type 2 papillae were observed less frequently, accounting for 8.7%. Among the 103 cases, 77 (74.76%) showed no PAD, 10 cases (9.71%) had Type 2 PAD, and 15 cases (14.56%) had Type 3 PAD. Type 1 PAD cases were excluded at the start of the study. No significant differences were observed in age, gender, indication for ERCP, or the prevalence of PAD across the four papilla types.

The total TPS success rate was 85.44%. Failure rates for TPS across the four papilla types—Types 1, 2, 3, and 4—were observed at 10.53%, 0%, 16.67%, and 28%, respectively. In all 103 cases, the average number of pancreatic duct wire passages exceeded five. Pancreatic duct injection occurred in 80 cases (76.67%), and the average cannulation time across these cases was over 26 min. There were no significant differences among the four papilla types in terms of the number of guidewire passages into the main pancreatic duct, contrast injection into the main pancreatic duct, or total cannulation time. In this study, the following procedures were performed: needle-knife sphincterotomy in 26 cases (25.24%), EPBD in 4 cases (3.88%), ERPD in 78 cases (75.73%), and stone extraction in 49 cases (47.57%). ERBD was conducted in 45 cases (43.69%), and lithotripsy was performed in 4 cases (3.88%). Subsequent needle-knife precut sphincterotomy procedures were performed on 26 patients (25.24%) because of the difficulty of succeeding with TPS alone. Similar therapeutic interventions, including needle-knife sphincterotomy, EPBD, ERPD, ERBD, stone extraction, and lithotripsy, were performed across the four papilla types.

The complication rates were 2.91% for perforation, 15.53% for PEP, 1.94% for bleeding, and 1.94% for cholangitis. Type 4 papilla showed a higher overall complication rate (32%) and a higher incidence of PEP (20%), but these were not statistically significant (Table 1).

We analyzed the rates of cannulation success and failure, as detailed in Table 2. There was a notable difference in age between the success and failure groups (*p* = 0.004). As presented in Table 3, both univariate and multivariate analyses indicated that age is a significant risk factor for cannulation failure. In the univariate analysis, Type 4 papilla exhibited a greater rate of cannulation failure compared to Type 1 and Type 2 papilla, reaching statistical significance (OR 3.89, *p* = 0.028). However, this distinction did not maintain statistical significance in the multivariate analysis (OR 3.58, *p* = 0.052). To ensure model stability, papilla Types 1 and 2 were grouped together because of the absence of failures in Type 2 cases, which could have led to instability in the regression model.

## 4. Discussion

In our investigation, 103 cases undergoing therapeutic ERCP were included. The papilla was classified according to Haraldsson’s endoscopic classification system [19]. All patients underwent TPS because of challenges in biliary cannulation. In instances in which TPS failed to achieve successful cannulation, another pre-cut procedure involving a needle knife may be implemented. In the univariate analysis, Type 4 papilla exhibited a higher cannulation failure rate compared to Types 1 and 2, and this finding was statistically significant. However, this distinction did not maintain statistical significance in the multivariate analysis. Moreover, age was identified as an independent risk factor for failure of TPS.

Successful cannulation of the biliary duct constitutes a crucial step in ERCP. Previous studies have suggested that papilla morphology may impact the success rate of biliary cannulation. Haraldsson developed a classification system that categorizes papilla into four types. He noted that small Type 2 and protruding or pendulous Type 3 papillae are often more difficult to cannulate [19,20]. Chen et al. similarly reported that small papillae and protruding or pendulous papillae pose greater difficulties in cannulation compared to regular papillae [15]. However, to date, there has been no investigation into whether papilla morphology affects the success rate of TPS. Our study represents the first attempt to address and discuss this specific issue.

In our study, the overall success rate for TPS was found to be 85.44%, aligning closely with findings in published studies [11,21,22]. In our previous investigation about the successful rate of TPS, there is a 46.15% failure rate of TPS in cases involving Type 4 papilla. Multivariate analysis further identified Type 4 papilla as an independent risk factor for TPS failure [18]. However, since 2023, our success rate has improved with the inclusion of new data, reducing the failure rate for Type 4 papilla cases to 28%. We included 46 new cases, among which 11 were identified as Type 4 duodenal major papilla. Out of these 11 cases, only one experienced cannulation failure, resulting in a failure rate of 9% for the new cases. This has reduced the overall failure rate from 46.15% to 28%. While Type 4 papilla exhibited a greater failure rate in the univariate analysis, this discrepancy was not statistically significant in the multivariate analysis (*p =* 0.052).

This improvement can be attributed to the growing familiarity and refinement of TPS techniques over time. As operators at our institution gained more experience, particularly in managing challenging papilla types like Type 4, the success rate increased significantly. Thus, while the current study’s statistical results do not show a significant difference, this likely reflects the impact of technical advancements and knowledge accumulation. In centers with less experience, Type 4 papilla may still present higher failure rates, highlighting the importance of continuous technique refinement.

**Figure 3:**
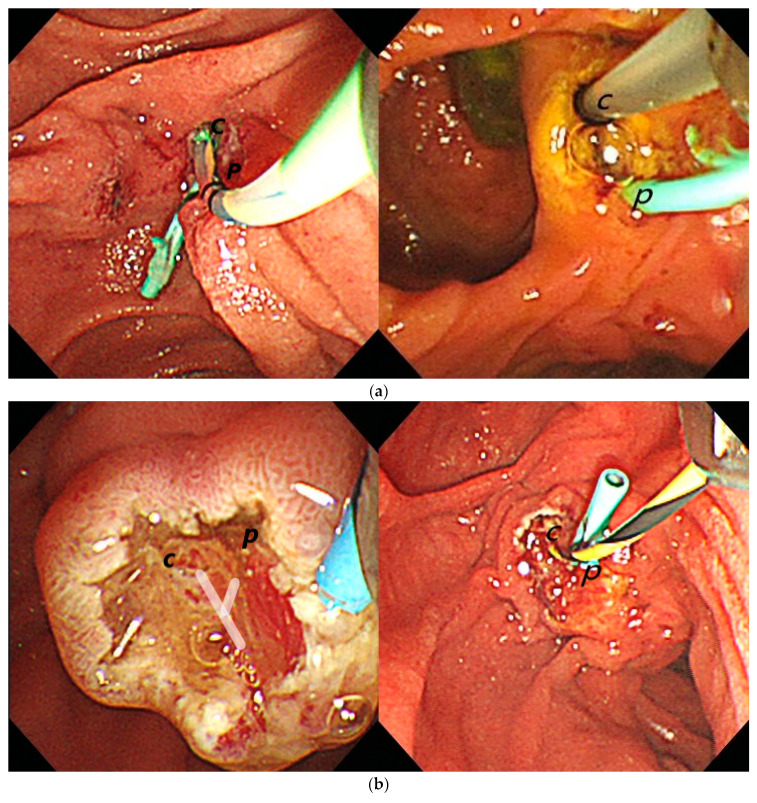

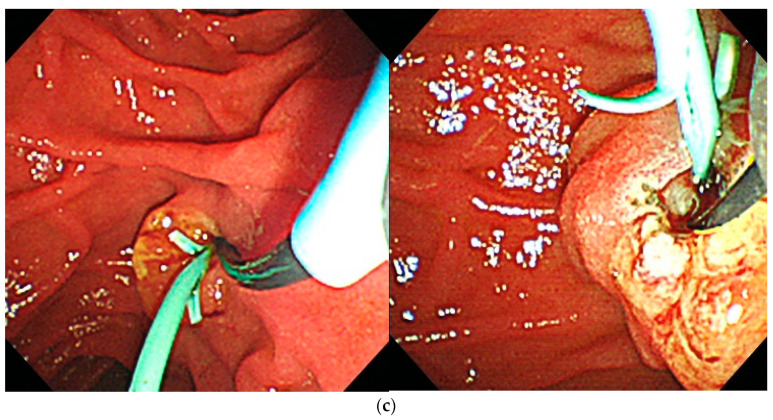
Biliary–pancreatic duct union (BPDU) patterns. (**a**) In Type A biliary–pancreatic duct union (BPDU) patterns, the ducts have a more extended union, allowing for a clear separation of the bile and pancreatic ducts following a full-cut transpancreatic sphincterotomy (TPS). (c, bile duct orifice; p, pancreatic duct orifice). (**b**) In Type B biliary–pancreatic duct union (BPDU) patterns, the ductal union is shorter. Bile duct orifice could align directly over the sphincter muscle. (c, bile duct orifice; p: pancreatic duct orifice). (**c**) In Type C biliary–pancreatic duct union (BPDU) patterns, the bile and pancreatic ducts remain distinct and are embedded within the sphincter muscle.

Optimizing TPS success requires a clear understanding of its reliance on biliary–pancreatic duct union (BPDU) pattern recognition, setting it apart from other techniques like double guidewire and needle-knife sphincterotomy. Success rates can be compromised, especially among beginners, when repeated cannulation attempts are made without considering the underlying ductal anatomy. Each BPDU pattern calls for a specific approach: Type A patterns, with long ductal unions, generally facilitate easier cannulation following complete duct separation through TPS. Type B patterns, featuring short unions, demand greater precision due to the bile duct orifice’s proximity to the sphincter muscle, while Type C patterns, where ducts remain separate within the sphincter, are the most challenging and may require supplementary needle-knife papillotomy (Figure 3). Further research is needed to validate TPS strategies tailored to specific papilla types, particularly Type 4, where BPDU variability may be higher than in other types and could influence success rates. This investigation is especially relevant for centers that have newly adopted TPS and for verifying the relationship between BPDU patterns and procedural outcomes. As Wang et al. demonstrated, adapting cannulation techniques to BPDU variations is crucial for TPS success [23]. These BPDU variations highlight the importance of strategies that consider both the papilla’s anatomical and morphological features, which could further improve TPS outcomes and reduce complications. An enhanced understanding of papilla morphology and BPDU patterns is essential for optimizing the results in complex cannulation cases.

Our investigation identified age as a significant risk factor for cannulation failure, consistent with findings from previous studies [24,25]. Emre et al. reported a 1.01-fold increase in the failure rate of cannulation for each one-year increment in patient age [24]. Sabbah et al. identified several independent predictors for failure in standard endoscopic techniques, including age over 65, the presence of an intra-diverticular papilla, a common bile duct diameter greater than 15 mm, and common bile duct stenosis [25]. A possible explanation for the increased difficulty of cannulation with advancing age is the duodenal distortion that can result from conditions like ulcers or cholangitis, making it hard to maintain the ideal approach angle to the papilla. Older patients, who may experience higher morbidity and frailty, also face a greater risk of TPS complications, potentially raising the likelihood of failure. Additionally, advanced age may correlate with an increased prevalence of PAD, further impacting TPS outcomes. In addition, PAD size may increase with age. Large PAD can obscure the papilla and alter its orientation, is often linked with cannulation failure. In our study, Type 1 PAD was excluded due to its influence on papilla classification.

The post-ERCP complication rates observed in our study were similar to those reported in prior research [11,21]. Despite conducting a comprehensive analysis, no specific risk factors for post-ERCP complications were identified within our cohort. This may underestimate the complexity of factors influencing post-procedural outcomes and suggests that further research may be necessary to delineate potential risk factors in this context.

In cases of difficult cannulation at our hospital, we currently do not use endoscopic ultrasound-guided rendezvous (EUS-RV). Instead, we rely on techniques like the double-guidewire method, TPS, needle-knife precut papillotomy, and fistulotomy. However, EUS-RV is worth considering as a future addition. Recent studies indicate that both EUS-RV and precut sphincterotomy achieve comparable success rates as salvage techniques for challenging bile duct cannulation in benign biliary disease, with complication rates that remain within acceptable limits [26]. TPS enhances cannulation success, but its potential complications (PEP, stenosis, and fibrosis) warrant careful consideration during follow-up procedures. The TPS-altered anatomy facilitates subsequent interventions, enabling easier SEMS placement (though requiring precise stent selection) and more efficient stone extraction through the enlarged opening, often reducing the need for mechanical lithotripsy.

This study has several limitations. First, it is a retrospective, single-center study with a relatively limited patient population. This limitation constrains the generalizability of the findings, and future studies involving larger, multicenter cohorts may be needed to strengthen the conclusions. Second, the classification of papilla is not always the same among different endoscopists, introducing the potential for interobserver variability. However, according to our report, the interobserver agreement was good in our institution [15]. Third, biliary cannulation was often initially performed by a fellow rather than a seasoned specialist at our institution. The lack of recorded cannulation times for both fellows and seasoned specialists is a notable limitation, potentially impacting the assessment of the cannulation success rate. However, it is noteworthy that our rate of failed TPS was 15%, comparable to previous studies [11,21,22]. Prior studies have suggested that trainee involvement may extend procedure duration; however, it does not appear to correlate with a higher incidence of immediate adverse events or technical failure [27]. The overall influence on the success rate appears to be acceptable given these considerations.

## 5. Conclusions

Our study indicates that the morphology of the major duodenal papilla may influence TPS success rates. Specifically, Type 4 papillae showed a higher cannulation failure rate than Type 1 and Type 2, reaching statistical significance in the univariate analysis, although this was not sustained in the multivariate analysis. Further studies are needed to confirm these observations. Adapting TPS techniques to accommodate particular papillae morphologies may enhance procedural outcomes. Additionally, age emerged as a significant, independent predictor of TPS failure.

## Figures and Tables

**Table 1:** Patient demographics by papilla type.

Total (*n =* 103)		Ampulla Vater Type				*p*-Value
		1 (*n =* 57)	2 (*n =* 9)	3 (*n =* 12)	4 (*n =* 25)	
Gender						0.507
Female	44 (42.72%)	21 (36.84%)	4 (44.44%)	7 (58.33%)	12 (48.00%)	
Male	59 (57.28%)	36 (63.16%)	5 (55.56%)	5 (41.67%)	13 (52.00%)	
Age	64.46 ± 15.67	62.53 ± 14.69	68.56 ± 16.52	67.83 ± 13.62	65.76 ± 18.47	0.364
Indication						0.745
Stone	57 (55.34%)	30 (52.63%)	7 (77.78%)	5 (41.67%)	15 (60.00%)	
Benign	10 (9.71%)	6 (10.53%)	0 (0%)	1 (8.33%)	3 (12.00%)	
Cancer	36 (34.95%)	21 (36.84%)	2 (22.22%)	6 (50.00%)	7 (28.00%)	
PAD						0.922
0	77 (74.76%)	41 (71.93%)	6 (66.67%)	11 (91.67%)	19 (76.00%)	
2	10 (9.71%)	7 (12.28%)	1 (11.11%)	0 (0%)	2 (8.00%)	
3	15 (14.56%)	8 (14.04%)	2 (22.22%)	1 (8.33%)	4 (16.00%)	
Failed cannulation	15 (14.56%)	6 (10.53%)	0 (0%)	2 (16.67%)	7 (28.00%)	0.118
Pancreatic duct wire passage	5.81 ± 3.41	5.96 ± 3.71	4.44 ± 2.51	6.83 ± 3.38	5.44 ± 2.93	0.463
Pancreatic duct injection	80 (77.67%)	44 (77.19%)	6 (66.67%)	9 (75.00%)	21 (84.00%)	0.689
Cannulation time (minute)	26.61 ± 15.32	27.08 ± 18.52	25.89 ± 10.54	27.38 ± 10.66	25.44 ± 10.27	0.867
Cannulation time (minute)						0.815
<10	5 (4.85%)	3 (5.26%)	0 (0%)	1 (8.33%)	1 (4.00%)	
10–20	23 (22.33%)	13 (22.81%)	3 (33.33%)	1 (8.33%)	6 (24.00%)	
20–30	28 (27.18%)	18 (31.58%)	1 (11.11%)	4 (33.33%)	5 (20.00%)	
>=30	47 (45.63%)	23 (40.35%)	5 (55.56%)	6 (50.00%)	13 (52.00%)	
NKS	26 (25.24%)	14 (24.56%)	2 (22.22%)	5 (41.67%)	5 (20.00%)	0.560
EPBD	4 (3.88%)	3 (5.26%)	1 (11.11%)	0 (0%)	0 (0%)	0.368
ERPD	78 (75.73%)	43 (75.44%)	7 (77.78%)	7 (58.33%)	21 (84.00%)	0.412
Stone extraction	49 (47.57%)	27 (47.37%)	7 (77.78%)	4 (33.33%)	11 (44.00%)	0.231
ERBD	45 (43.69%)	25 (43.86%)	4 (44.44%)	6 (50.00%)	10 (40.00%)	0.954
Lithotripsy	4 (3.88%)	2 (3.51%)	1 (11.11%)	0 (0%)	1 (4.00%)	0.529
Complication	22 (21.36%)	13 (22.81%)	1 (11.11%)	0 (0%)	8 (32.00%)	0.121
Bleeding	2 (1.94%)	0 (0%)	0 (0%)	0 (0%)	2 (8.00%)	0.197
PEP	16 (15.53%)	11 (19.30%)	0 (0%)	0 (0%)	5 (20.00%)	0.218
Cholangitis	2 (1.94%)	1 (1.75%)	0 (0%)	0 (0%)	1 (4.00%)	0.696
Perforation	3 (2.91%)	2 (3.51%)	1 (11.11%)	0 (0%)	0 (0%)	0.307

Kruskal–Wallis test. Fisher’s exact test. Continuous data were reported as mean ± SD. Categorical data are presented as numbers and percentages. Haraldsson’s classification was utilized to categorize the papillae morphologies into four types: regular (Type 1), small (Type 2), protruding or pendulous (Type 3), and creased or ridged (Type 4). PAD, periampullary diverticulum; NKS, needle-knife sphincterotomy; ERPD, endoscopic retrograde pancreatic drainage; EPBD, endoscopic papillary balloon dilation; ERBD, endoscopic retrograde biliary drainage; PEP, post-endoscopic retrograde cholangiopancreatography pancreatitis.

**Table 2:** Comparison of transpancreatic sphincterotomy success vs. failure.

	Success (*n =* 88)	Failure (*n =* 15)	*p*-Value
Papilla type			0.069
1 + 2	60 (68.18%)	6 (40.00%)	
3	10 (11.36%)	2 (13.33%)	
4	18 (20.45%)	7 (46.67%)	
Papilla type			0.118
1	51 (57.95%)	6 (40.00%)	
2	9 (10.23%)	0 (0%)	
3	10 (11.36%)	2 (13.33%)	
4	18 (20.45%)	7 (46.67%)	
Gender			0.738
Female	37 (42.05%)	7 (46.67%)	
Male	51 (57.95%)	8 (53.33%)	
Age	62.63 ± 15.15	75.20 ± 14.72	0.004 **
Indication			0.722
Stone	48 (54.55%)	9 (60.00%)	
Benign	8 (9.09%)	2 (13.33%)	
Cancer	32 (36.36%)	4 (26.67%)	
PAD			0.521
0	67 (76.14%)	10 (66.67%)	
2 + 3	21 (23.86%)	5 (33.33%)	

Mann–Whitney U test, Chi-Square test, and Fisher’s exact test. ** *p* < 0.01. Continuous data are reported as the mean ± SD. Categorical data are presented as numbers and percentages. PAD, periampullary diverticulum.

**Table 3:** Risk factors for transpancreatic sphincterotomy failure.

	Univariate			Multivariate		
	OR	(95% CI)	*p*-Value	OR	(95% CI)	*p*-Value
Age	1.07	(1.02–1.12)	0.006 **	1.06	(1.01–1.11)	0.011 *
Gender						
Female	1.00					
Male	0.83	(0.28–2.49)	0.738			
Indication						
Stone	1.00					
Benign	1.33	(0.24–7.34)	0.741			
Cancer	0.67	(0.19–2.35)	0.528			
Papilla type						
^!^ 1 + 2	1.00			1.00		
3	2.00	(0.35–11.33)	0.434	1.72	(0.29–10.24)	0.549
4	3.89	(1.16–13.05)	0.028 *	3.58	(0.99–12.92)	0.052
PAD						
0	1.00					
2 + 3	1.60	(0.49–5.19)	0.438			

Logistic regression. * *p* < 0.05 and ** *p* < 0.01. PAD, periampullary diverticulum. ^!^ Papilla Types 1 and 2 are grouped together due to the absence of failures for Type 2 cases, which could cause instability in the statistical model. Grouping these types ensured better model performance and interpretability.

## Data Availability

Data supporting the findings of this study are accessible from the corresponding author upon reasonable request.

## Appendix A

**Table A1:** Interobserver agreement evaluation.

Cohen’s Weighted Kappa					
Weighted Kappa	Asymptotic			95% Asymptotic Confidence Interval	
	Std. Error	zc	Sig.	Lower Bound	Upper Bound
0.963	0.024	11.603	<0.001	0.916	1.010
